# Multiplexed single-cell and spatial profiling reveal B cells and tertiary lymphoid structures as prognostic indicators in pleural mesothelioma

**DOI:** 10.1038/s41416-026-03421-1

**Published:** 2026-04-27

**Authors:** Angelica Rigutto, Nicolás G. Núñez, Jenny C. Kienzler, Isabelle Opitz, Mayura Meerang, Martina Haberecker, Nadine Fournier, Joao Lourenço, Raphael Gottardo, Karina Silina, Anurag Gupta, Burkhard Becher, Alessandra Curioni-Fontecedro

**Affiliations:** 1 Department of Medical Oncology and Hematology, University Hospital Zurich, Zurich, Switzerland; 2 Institute of Experimental Immunology, University of Zurich, Zurich, Switzerland; 3 Department of Thoracic Surgery, University Hospital Zurich, Zurich, Switzerland; 4 Department of Pathology and Molecular Pathology, University Hospital Zurich, Zurich, Switzerland; 5 Translational Data Science Facility, Swiss Institute of Bioinformatics, Agora Cancer Research Center, Lausanne, Switzerland; 6 Institute of Pharmaceutical Sciences, Department of Chemistry and Applied Biosciences, ETH Zurich, Zurich, Switzerland; 7 Faculty of Science and Medicine, University of Fribourg, Fribourg, Switzerland; 8 Present Address: Faculty of Science and Medicine, University of Fribourg, Fribourg, Switzerland; 9 Present Address: Facultad de Ciencias Químicas, Departamento de Bioquímica Clínica, Universidad Nacional de Córdoba, Córdoba, Argentina; 10 Present Address: Department of Oncology, Cantonal Hospital Fribourg, Villar sur Glâne, Switzerland

**Keywords:** Cancer microenvironment, Lymphoid tissues

## Abstract

**Background:**

Pleural mesothelioma (PM) is an orphan disease with poor prognosis. While T cell dynamics in the tumor microenvironment (TME) have been extensively studied, the role of B cells remains poorly characterized. Tumor-infiltrating B cells, particularly when organized into tertiary lymphoid structures (TLS), have been associated with improved outcomes of patients with cancer.

**Methods:**

In this study, high-dimensional flow cytometry (HDCyto) and high-plex imaging were applied to analyze fresh-frozen and formalin-fixed paraffin-embedded (FFPE) PM tumor samples, enabling a comprehensive immune profiling of the TME.

**Results:**

We identified 15 distinct immune cell subsets and stratified tumors into three subgroups with significantly different survival outcomes. Longer survival correlated with increased T and B cell infiltration, with B cells and CD4+ T cells forming TLS in specific cases.

**Conclusions:**

These findings underscore the heterogeneity of PM tumors and highlight the critical role of B cells and TLS in shaping anti-tumor immunity and influencing patient prognosis.

## Introduction

Pleural mesothelioma (PM) is an aggressive tumour most frequently caused by asbestos exposure, with a poor prognosis, that develops in the pleura, the thin membrane surrounding the lung. Asbestos exposure is the primary cause of PM development, leading to its ban in developed countries. While PM cases are declining in Western countries [1], the frequent use of asbestos in developing nations is likely to drive an overall increase in global incidence rates [2]. Because intrinsic resistance to most therapies occurs, few treatment options are available for patients with PM. The primary approach is systemic treatment with platinum-based chemotherapy, which provides only short-term efficacy and fails to improve long-term survival [3, 4]. Immunotherapies, especially immune checkpoint inhibitors (ICI) have shown meaningful clinical response for multiple cancer types [5]. The use of ICI to treat PM has improved survival of PM patients compared to standard chemotherapy [6–8], but radiological responses occur in the minority of cases [9]. Therefore, it is crucial to investigate how the immune system can interfere with cancer growth to understand unresponsiveness and resistance to ICI in these patients [10].

The tumour microenvironment (TME) plays a critical role in shaping response and resistance to cancer treatments [11]. The TME is a dynamic, complex and heterogeneous milieu composed of multiple cell types that include tumour cells, immune cells such as T and B lymphocytes, macrophages, dendritic cells, natural killer (NK) cells and stromal cells, endothelial cells, and fibroblasts. The ICIs aim to affect the TME, where they influence cellular crosstalk between immune cells and cancer cells, crucial for both disease progression and response to therapy. In this context, the role of CD8^+^ T cells, CD4^+^ T cells and NK cells has been explored to a greater extent, revealing a correlation between their presence and patient outcomes [12–15]. However, the impact of other immune cells, such as B cells, has been insufficiently studied for this disease. Recent studies indicate that tumour-infiltrating B cells play a pivotal role in anti-tumour responses and are associated with improved outcomes [16–18]. In chronically inflamed tissues, such as tumours, B cells are localized within dense immune cell niches known as tertiary lymphoid structures (TLS). The formation of TLS is a dynamic, multi-stage process that begins with unstructured dense immune cell niches and evolves into a fully developed state capable of supporting germinal centre formation [19]. Their presence and maturation were shown to support anti-tumour immunity and strongly correlate with patient outcomes [20–23].

In this study, we sought to elucidate the cellular composition and complexity of the TME in pleural mesothelioma (PM) through comprehensive immune profiling. Employing two high-dimensional single-cell technologies—spectral flow cytometry (HDCyto) and high-plex imaging—we conducted an in-depth characterisation of immune cell populations within PM tumours. This analysis enabled the classification of tumours into three distinct subgroups, each associated with significant differences in patient survival. Notably, B cells and CD4^+^T cells in patients with favourable outcomes were preferentially organized into TLS, suggesting a critical role for these immune architectures in shaping anti-tumour immunity and influencing disease prognosis.

## Material and methods

### Patient cohort

From 2017 to 2021, a total of 54 PM patients were included in the study. From all patients, a tumour sample was collected during surgery or diagnostic biopsy at the University Hospital Zurich. Among these, 7 patients were excluded following diagnosis review, and 6 were excluded due to poor data quality [24]. In total, 41 samples were analysed. All tumour samples were analysed as fresh-frozen tissue to study the abundance of immune cell subsets. Additionally, 30 matching FFPE tumours were analysed to investigate TLS and the spatial distribution of TLS-associated immune cells to define TLS stages. Clinical data are summarized in Table 1 and extend data is available in Supplementary Table 4.

**Table 1:** Patient’s characteristics showing gender distribution, classification of the disease based on histology and other clinical parameters.

Patient’s characteristics	Total patients (*n* = 41)
Gender (%)	
Male	39 (95.1)
Female	2 (4.9)
Age at diagnosis (mean), years (range)	68.4 [53–83]
Histology (%)	
Epithelioid	37 (90.2)
Sarcomatoid	1 (2.5)
Biphasic	3 (7.3)
Asbestos exposure	
Yes	28 (68.3)
Possible	10 (24.4)
No	3 (7.3)
Chemotherapy (%)	
Yes	28 (68.3)
No	13 (31.7)

All patients included in the study gave their written informed consent. The study was performed according to the Declaration of Helsinki and was approved by the cantonal ethics committee (EK-ZH-2020-02566).

### Tumor samples preparation

Tumour tissue was cut into pieces, frozen in freezing medium (10% DMSO/FCS), and stored as fresh-frozen specimens in liquid nitrogen until further use. Thawed tumour samples were digested using the Tumour Dissociation Kit (Miltenyi Biotec) according to the manufacturer’s instructions. Briefly, tumour pieces were incubated with the enzymatic solution in RPMI 1640 at 37 °C for 90 minutes with continuous shaking. Single-cell suspension of dissociated tissues was filtered and smashed through a 100 µm cell strainer and RPMI 1640 (10% FBS, 2 mM L-glutamine, 50 units Penicillin, 50 µg/mL Streptomycin, 25 mM HEPES) was added to stop the enzymatic reaction. Cells were washed with phosphate-buffered saline (PBS) and centrifuged at 400 × *g* for 5 minutes at 4 °C to obtain the pellet.

### Formalin-fixed paraffin embedded (FFPE) tissue preparation

FFPE PM tissues were sectioned at 4 μm thickness using a rotary microtome and mounted on Superfrost Plus microscope slides (Thermo Fisher Scientific). Slides were dried at room temperature (RT) and baked at 60 °C overnight. Tissue sections were deparaffinized with two washes of xylene, rehydrated through a graded ethanol series with two washes each (100%, 95%, 70%, and 50%) and incubated in PBS for 10 minutes, followed by incubation in 0.3% Triton X-100 for 10 minutes and washing in PBS. Tissue sections were subjected to a two-step antigen retrieval procedure in citrate-based antigen unmasking solution at pH 6 (Vector Labs) followed by incubation in Tris-antigen retrieval solution (100 mM Tris base, 10 mM EDTA, 0.5% Tween 20, pH 9). Slides were blocked with 10% donkey serum (Jackson Immuno) for 1 hour at RT, followed by autofluorescence quenching with a high-power LED (Samsung). The staining for high-plex imaging was performed in a semi-automatic manner, where a technician prepared the solution, added the antibody mix manually, while washing steps were performed by a robot.

### High-dimensional spectral flow cytometry staining

Single-cell suspensions obtained from the digested tumour samples were stained with Live/Dead Zombie NIR (Biolegend) in PBS for 10 minutes at RT in the presence of Human TruStain FcX (Fc Receptor Blocking Solution; Biolegend). Without washing, the cells were spun down, resuspended in the cell surface antibody mixture in PBS (Supplementary Table 1), and incubated at 4 °C for 30 minutes. After surface staining, cells were washed with PBS and fixed using the fixation buffer from Foxp3/Transcription Factor Staining Buffer Set (eBioscience) for 45 minutes at 4 °C according to the manufacturer’s instructions. Subsequently, cells were washed once with permeabilization buffer (PB) from the same kit and incubated with the intracellular antibody mixture in PB for 30 minutes at 4 °C (Supplementary Table 1). After incubation, cells were washed with PB and centrifuged at 800 × *g* for 3 minutes. Cells were acquired using the spectral analyser Cytek Aurora (Cytek Biosciences).

### High-plex imaging staining of immune cells in TLS

Antibodies used to characterise the immune cells within TLS and their maturation stage are listed in Supplementary Table 1. The assessment of TLS maturation stages was performed as follow: early TLS (E-TLS) with dense immune cell niches expressing CD20 and CD4, but lacking follicular dendritic cells (CD21) and germinal centres (CD23); primary follicle-like TLS (PFL-TLS) with dense immune cell niches expressing CD20, CD4 and CD21 but lacking CD23; secondary follicle-like TLS (SFL-TLS) with dense immune cell niches expressing all CD20, CD4, CD21 and CD23.

Tissue sections were stained with DAPI (100 μg/mL, Thermo Fisher Scientific) for 15 minutes at RT. Antibodies staining was performed in sequential rounds, each followed by chemical quenching of fluorophores. Antibodies were incubated for 1 hour at RT in 0.3% Bovine Serum Albumin (BSA)/PBS, and the fluorophores were chemically quenched using hydrogen peroxide and 0.5 M NAHCO_3_ solution between the different staining rounds. Tissues were imaged at 20× magnification using the Cell Dive high-plex imager (Leica Microsystems). Images analysis was performed with Aivia software (Leica Microsystems).

The Densities of TLS on H&E slides were performed by a pathologist. Later tumour slides were stained for TLS-related markers (CD4, CD20, CD21 and CD23) and quantified in Qupath version 0.5.2 by manual annotation to complement and validate H&E analysis of total TLS density. Total density of annotated TLS was defined as clusters of at least 60 CD20^+^ B cells accompanied by CD4^+^ T cells inside or adjacent to the tumour tissue divided by biopsy area (mm^2^). The density of differentially matured TLS was calculated (Number of E-TLS/mm^2^, number of PFL-TLS/mm^2^ and number of SFL-TLS/mm^2^, respectively) and plotted per group, with TLS densities spanning from 0.0 (no TLS for respective maturation stage present) to approx. 0.6 (0.6 TLS per mm^2^ of biopsy tissue) for E-TLS, 0.0 to approx. 0.2 for PFL-TLS and 0.0 to approx. 0.09 for SFL-TLS.

### Spectral flow data pre-processing and quality control

Unmixed FCS files were preprocessed using FlowJo software version 10.8.1. Dead cells, doublets and cells stained by fluorochrome aggregates were excluded from the analysis. CD45^+^ cells were exported from FlowJo and imported into R version 4.2.0, using flowcore R package [25].

### Spectral flow data analysis

Logicle transformation was applied to the data using the *transform* function from the flowCore R package. Parameters were set to *m* = 6 (full width of the transformed display in asymptotic decades) and *t* = 4 × 10^6^ (top of the scale data value). Other parameter values were estimated using the *estimateLogicle* function. Further automatic quality control was performed using the *flow_auto_qc* function from the flowAI R package version 1.28.0 [24], after which only high-quality events were retained.

Data was sub-sampled to include 2000 CD45^+^ cells from each sample (with replacement) using a custom R function. Pseudobulk-level Multi-Dimensional Scaling (MDS) analysis was performed using the *pbMDS* function from the CATALYST R package. Non-Redundancy Scores (NRS) were computed by antigen using the *plotNRS* function from the CATALYST R package.

### Identification of immune cell populations from spectral flow cytometry data

To identify immune cell populations and ensure an unbiased analysis, we performed dimensionality reduction on cytometry data by applying Principal Component Analysis (PCA), followed by Uniform Manifold Approximation and Projection (UMAP) to systematically reduce the data to two dimensions and visualise cell distributions. UMAP was performed using the *runDR* function with default parameter values from the CATALYST R package [26]. Unsupervised clustering was applied to all cells using FlowSOM algorithm [27], in combination with the ConsensusClusterPlus algorithm [28] using the cluster function from the CATALYST R package. Clusters identified were subsequently manually annotated and merged based on median antigen expression.

### Statistical analysis and data availability

Summary of all analysed data and extended clinical data is available in supplementary data file.

### Differential abundance analysis

Manually annotated clusters were used to calculate the relative frequencies of immune cell populations. Differential abundance analysis between groups was performed using genewise negative binomial generalized linear models with quasi-likelihood tests, as implemented in the glmQLFit and *glmQLFTest* functions of the edgeR R package version 3.40.2 [29].

To compare the densities of TLS across the three groups, we applied the Kruskal-Wallis rank sum test, using the function *kruskal.test* from the R stats package. Pairwise comparison between subgroups was performed using the two-tailed Wilcoxon-signed rank sum test, as implemented in the *wilcox.test* function from the R stats package. *P* values were adjusted for multiple testing using the Benjamini–Hochberg method, as implemented in the *p.adjust* function from the R stats package.

### Survival analysis

Overall survival (OS) was defined as the time from the start of treatment to death or last follow-up. Progression-free survival (PFS) was defined as the time from start of treatment to date of first progression or last follow-up. Only patients with a known date of treatment initiation were included in the survival analysis. Kaplan–Meier survival curves were created using the *survfit* function from the survival R package version 3.5.5 [30] and visualised with the *ggsurvplot* function from the survminer R package [31]. A log-rank test was used to compare Kaplan–Meier survival curves. To assess the effect of quantitative predictor variables (risk factors) on OS, we used Cox proportional hazards regression analysis, as implemented in the *coxph* function from the survival R package. A multivariate analysis was performed to evaluate OS and PFS including the following factors as chemotherapy, surgery, gender and age.

## Results

### Identification of immune cell subsets by HD flow cytometry

**Fig. 1:**
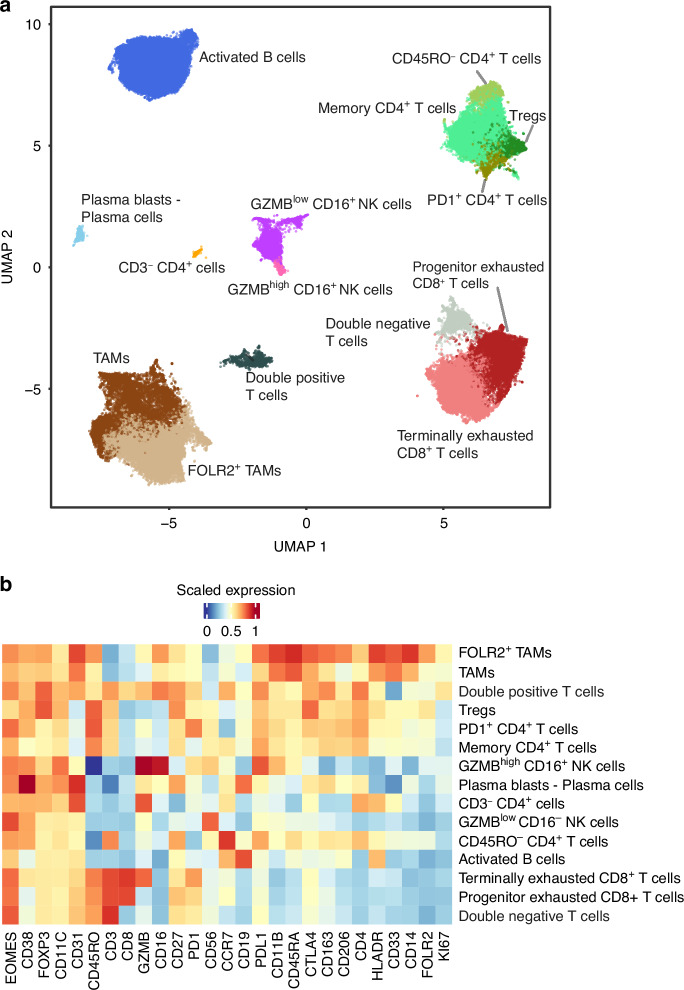
Identification of immune cell subsets infiltrating PM tumors. **a** UMAP map representing the FlowSOM-guided manual metaclustering of CD45^+^ cells. 15 different immune cell populations were identified. **b** Heatmap indicating the median scaled expression of markers used to manually annotate the populations represented in **a**.

41 cryopreserved tumour samples were analysed using HDCyto. To investigate the heterogeneity of the TME, we designed a multi-colour flow cytometry panel, analysing 30 surface markers and transcription factors (Supplementary Table 1). Fibroblast activation protein, mesothelin and CD31 were used to exclude fibroblasts, tumour cells and endothelial cells, respectively. To visualise each immune cell subset infiltrating the tumours, we generated a UMAP plot based on all 27 immune cell markers to compute estimated cell similarities. Unsupervised clustering of CD45^+^ cells resulted in the identification of 15 immune cell subsets (Fig. 1a), which were manually annotated based on the median marker intensities (Fig. 1b and Supplementary Fig. 1).

The T cell subpopulations were defined as terminally exhausted CD8^+^ T cells, progenitor exhausted CD8^+^ T cells, double negative T cells (CD3^+^ CD4^-^ CD8^-^), double positive T cells (CD3^+^ CD4^+^ CD8^+^), regulatory T cells (Tregs), PD1^+^ CD4^+^ T cells, CD3^-^CD4^+^ cells, memory CD4^+^ T cells and CD45RO^-^ CD4^+^ T cells. Terminally exhausted CD8^+^ T cells were characterised by medium/high expression of CD38, granzyme B (GZMB), CD45RO and inhibitory molecules such as PD1 and CTLA4. On the contrary, progenitor exhausted CD8^+^ T cells were characterised by low expression of CD38, GZMB, PD1 and CTLA4 [32]. The subpopulations of B and NK cells included activated B cells (HLADR^high^), plasma blasts/plasma cells (CD38^high^ HLADR^low^) and natural killer (NK) cells, namely GZMB^low^ CD16^-^ and GZMB^high^ CD16^+^ NK cells.

Two different myeloid cell subsets were characterised: tumour-associated macrophages (TAMs) and FOLR2^+^ TAMs. Tissue-resident macrophages were annotated based on the folate receptor 2 (FOLR2) marker expression [33].

### PM tumors cluster in three different subgroups based on the differential abundance of infiltrating immune cell subsets

**Fig. 2:**
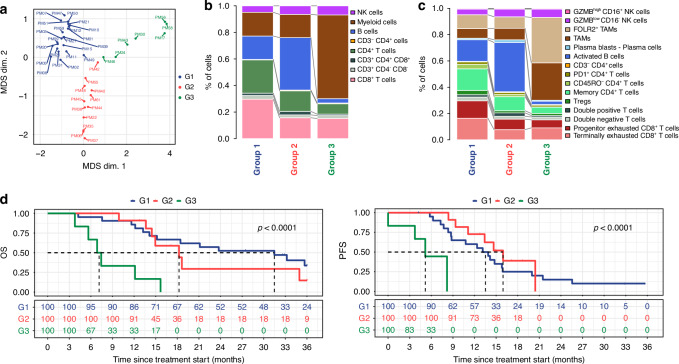
PM tumors cluster in three different subgroups that correlate with clinical outcome. **a** Multi-dimensional scaling plot showing PM tumors. Calculations were based on the median (logicle-transformed) marker expression of 27 leucocytes markers across all CD45^+^ cells measured for each sample, colored by group. Stacked bar plots representing the distribution of major immune cell populations (**b**) and immune cell subsets (**c**) infiltrating PM tumors divided by subgroup (Group 1, *n* = 22; Group 2, *n* = 12; Group 3, *n* = 7). **d** Survival analysis based on PM subgroups. The log-rank test was used to compare Kaplan–Meier survival curves.

To investigate similarities and differences within our cohort, we applied unbiased multidimensional scaling (MDS) to the total CD45^+^ cells. Interestingly, both dimensions highlighted a substantial heterogeneity among PM samples, revealing three distinct subgroups (group 1 = 22 samples; group 2 = 12 samples; group 3 = 7 samples) (Fig. 2a). When analysed further, major immune cell populations in group 1 and 2 were enriched in lymphoid cells such as B cells, CD4^+^ and CD8^+^ T cells, while group 3 mostly contained myeloid cells (Fig. 2b). In depth analysis of these major immune cell populations revealed differences among subsets of B, T and myeloid cells (Fig. 2c and Supplementary Fig. 2)

The clear distinction of three PM subgroups led us to examine their biological relevance; thus, survival analysis was performed. Univariate analysis demonstrated that patients in group 3 had a significantly worse median OS (mOS group 1: 31.9 months; mOS group 2: 18.5 months; mOS group 3: 7.3 months), and shorter median PFS (mPFS group 1: 13.7 months; mOS group 2: 16.2 months; mOS group 3: 5.2 months) compared to patients in group 1 and 2 (*p*-value < 0.0001). A multivariate analysis to evaluate OS and PFS, including the following factors as chemotherapy, surgery, gender and age showed that belonging to group three was the only significant prognostic factor for both OS and PFS with a very strong significant (Fig. 2d and Supplementary Table 2).

**Fig. 3:**
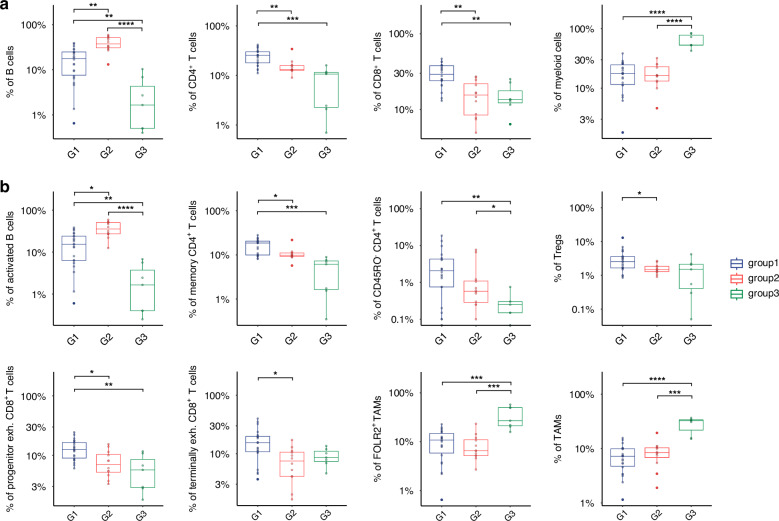
Differential abundance analysis of immune cells revealed differences between subgroups. Differential abundance analysis of major immune cell populations (**a**) and immune cell subsets (**b**) across the three subgroups (Group 1, *n* = 22; Group 2, *n* = 12; Group 3, *n* = 7) was performed. Only significant results are reported (*p* ≤ 0.05 (*), *p* ≤ 0.01 (**), *p* ≤ 0.001 (***), *p* ≤ 0.0001 (****); empirical Bayes quasi-likelihood *F*-test).

To further explore differences in these three subgroups, we performed differential abundance analysis of major immune cell populations and their subsets (Fig. 3a, b, Supplementary Table 3, and Supplementary Fig. 3). Group 1 showed a significantly increased infiltration of memory CD4^+^ T cells, CD45RO^-^ CD4^+^ T cells, Tregs, terminally exhausted CD8^+^ T cells and progenitor exhausted CD8^+^ T cells compared to group 2 and group 3. Interestingly, compared to group 3, B cells were more abundant in group 1 and group 2, with the highest presence in group 2. Group 3 showed an increased infiltration of TAMs and FOLR2^+^ TAMs.

### TLS presence characterizes tumors of PM patients with improved survival

**Fig. 4:**
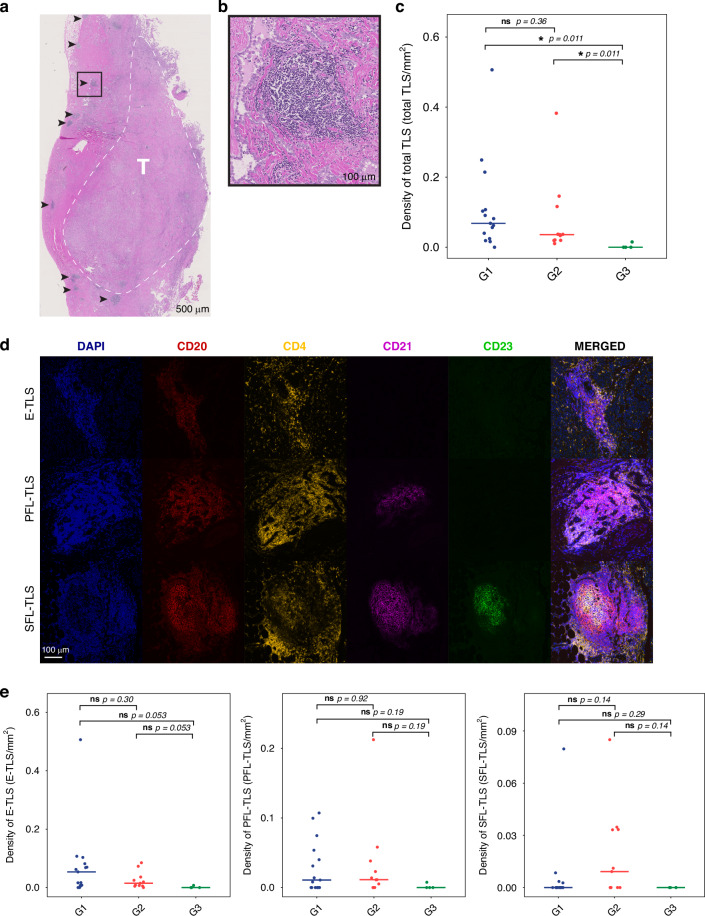
TLS structures characterise tumors from patients with longer survival. **a** Representative image of H&E-stained PM tumor showing multiple TLS in the normal tissue adjacent to the tumor (T). TLS are depicted with black arrows. Scale bar, 500 μm. **b** Higher magnification of one TLS depicted in **a**. **c** Comparison of total TLS density across subgroups (Group 1, *n* = 15; Group 2, *n* = 11; Group 3, *n* = 4. Wilcoxon-signed rank test). **d** Representative images of TLS in each maturation stage (E-TLS, PFL-TLS and SFL-TLS). CD20 for B cells, CD4 for T cells, CD21 for FDCs, CD23 for GC cells. **e** Comparison of TLS density at each maturation stage across subgroups (Group 1, *n* = 15; Group 2, *n* = 11; Group 3, *n* = 4. Wilcoxon-signed rank test). *p* ≤ 0.05 (*), *p* ≤ 0.01 (**), *p* ≤ 0.001 (***), *p* ≤ 0.0001 (****).

Based on the specific abundance of B cells and CD4^+^ T cells, particularly in Group 1 and 2, we explored the spatial distribution of these cells within the tumour and determined their association with the presence of TLS and their maturation stages. To do so, we performed high-plex imaging analysis on 30 matching FFPE tumour tissues (group 1 = 15; group 2 = 11; group 3 = 4). TLS were initially quantified in H&E-stained sections, followed by high-plex imaging analysis on Aivia software. We identified that the majority of TLS were present in the peritumoral areas (Fig. 4a, b). Tumors from patients in group 1 and 2 exhibited a significantly higher total TLS density compared to group 3 (Fig. 4c, Table 2, and Supplementary Fig. 4). Further, the different maturation stages were evaluated across all tumors (Fig. 4d). The density of differentially matured TLS was calculated (number of E-TLS/mm^2^, number of PFL-TLS/mm2 and number of SFL-TLS/mm^2^, respectively) and plotted per group (Fig. 4e), with TLS densities spanning from 0.0 (no TLS for respective maturation stage present) to approx. 0.6 (0.6 TLS per mm^2^ of biopsy tissue) for E-TLS, 0.0 to approx. 0.2 for PFL-TLS and 0.0 to approx. 0.09 for SFL-TLS. While no significant differences were observed in the densities of E-TLS, PFL-TLS and SFL-TLS among the groups (Fig. 4e), groups 1 and 2 exhibited a significantly higher absolute number of E-TLS compared to group 3 (Supplementary Fig. 4).

**Table 2:** Summary of the absolute counts and density of total TLS, as well as TLS at different maturation stages.

		E-TLS		PFL-TLS		SFL-TLS		Total TLS	
ID	Group	Absolute count	Density	Absolute count	Density	Absolute count	Density	Absolute count	Density
PM01	G1	0	0	0	0	0	0	0	0
PM02	G1	1	0.107	1	0.107	0	0	2	0.214
PM03	G1	4	0.506	0	0	0	0	4	0.506
PM05	G1	6	0.017	11	0.031	3	0.008	20	0.056
PM07	G1	2	0.082	0	0	0	0	2	0.082
PM08	G1	7	0.070	10	0.100	8	0.080	25	0.249
PM09	G1	2	0.005	4	0.011	1	0.003	7	0.019
PM11	G1	0	0	1	0.040	0	0	1	0.040
PM12	G1	1	0.068	0	0	0	0	1	0.068
PM21	G1	1	0.008	1	0.008	0	0	2	0.016
PM26	G1	1	0.103	0	0	0	0	1	0.103
PM27	G1	2	0.054	2	0.054	0	0	4	0.107
PM31	G1	10	0.062	0	0	0	0	10	0.062
PM49	G1	5	0.016	23	0.075	0	0	28	0.091
PM50	G1	2	0.007	4	0.014	1	0.004	7	0.025
PM06	G2	1	0.005	1	0.005	0	0	2	0.010
PM35	G2	5	0.036	0	0	0	0	5	0.036
PM36	G2	2	0.007	4	0.014	0	0	6	0.021
PM37	G2	3	0.008	4	0.011	0	0	7	0.020
PM40	G2	4	0.015	3	0.011	3	0.011	10	0.037
PM42	G2	3	0.025	7	0.058	4	0.033	14	0.116
PM44	G2	0	0	0	0	1	0.033	1	0.033
PM45	G2	21	0.073	11	0.038	10	0.035	42	0.146
PM48	G2	1	0.005	5	0.023	2	0.009	8	0.037
PM55	G2	1	0.019	0	0	0	0	1	0.019
PM61	G2	6	0.085	15	0.212	6	0.085	27	0.382
PM17	G3	0	0	0	0	0	0	0	0
PM30	G3	1	0.008	1	0.0076	0	0	2	0.015
PM43	G3	0	0	0	0	0	0	0	0
PM56	G3	0	0	0	0	0	0	0	0

## Discussion

This study provides an important conceptual advance in our understanding of the TME in PM and its role in dynamic cancer development. We identified that the leukocyte compartment within the TME of PM can be characterised into three different subgroups, which may help treatment outcome expectations. Moreover, we found that TLS characterised tumours from patients with improved survival, highlighting their potential as prognostic biomarkers.

Frequent unresponsiveness and development of resistance to treatments remain a clinical challenge when treating cancer patients. The dynamic nature of cancer tissue favours tumour growth by hijacking key physiological processes that are normally essential for healthy tissue function. Treatment with ICI has revolutionized cancer therapy, but not all tumour entities respond equally well, with some patients achieving prolonged benefits and others experiencing transient effects. The success of these therapies relies on tumour-related features such as the mutational landscape [34] and PD-L1 expression [35], as well as features of TME [36]. For the past two decades, numerous studies have focused on characterising the TME, revealing that its cellular composition can significantly influence treatment response. However, the dynamic changes within the TME often lead to treatment resistance, rendering therapies ineffective after an initial response. A deeper characterisation of the TME, particularly the infiltrating immune cell subsets, is therefore essential to develop more effective therapeutic approaches and to refine current treatment modalities.

While the TME of common cancer types such as lung, breast, and colon cancer has been extensively studied, our understanding of the TME in rare cancers such as PM remains limited. Recent research has made progress in characterising the PM TME; however, a detailed exploration of its cellular composition, particularly at the immune cell level, is still scarce. In this study, we aim to provide a comprehensive characterisation of tumour-associated immune cells within the PM TME using high-dimensional single-cell technologies. By enhancing current knowledge of PM TME, our findings may contribute to improving future therapeutic approaches for this lethal disease.

The immune cell-level analysis significantly categorised PM patients into three subgroups based on the abundance of specific immune cell subsets. Notably, we found that patients in groups 1 and 2 exhibited an enrichment of T and B cell subsets- Particularly Group 1 was enriched with memory CD4^+^ T cells, CD45RO^-^ CD4^+^ T cells, Tregs, terminally exhausted CD8^+^ T cells and progenitor exhausted CD8^+^ T cells. B cells were more abundant in group 1 and group 2, with the highest presence in group 2. Notably, Group 3 lacked lymphoid cell population and was enriched in myeloid cells, where TAMs and FOLR2^+^ TAMs subsets were enriched (Supplementary Table 3).

Our results align with the classification proposed by Patil and colleagues, who identified three different immune subgroups [37]. Matching to their study defining two immune-inflamed subgroups, characterised by varying levels of B and T cells-related gene expression, we show that Groups 1 and 2 have similar signatures for T and B cells. However, Patil et al. described the third subset as an immune-desert microenvironment, most likely due to lack of T and B cells. In this study, we did not classify Group 3 as an immune desert; we found that Group 3 is rather enriched with myeloid cells and lacking T and B cells. Such signatures can define groups 1 and 2 as immune-inflamed, while Group 3 as immune-suppressed.

Several studies have reported that the composition of the TME can have an impact on patients’ clinical outcomes and survival. The presence of B cells and T cells in the TME is associated with better outcomes in PM [13, 15, 38, 39], while TAMs negatively correlated with survival [40–42]. Our findings in PM appear supported by work in different oncology fields, demonstrating the significant role of immune cell subsets for OS and PFS, where patients with tumours enriched in myeloid cells (Group 3) showed a worse outcome compared to groups 1 and 2.

In recent years, there has been increasing interest in investigating the role of B cells in the context of anti-tumour immune responses. Along with CD4^+^ T cells, B cells are key components of TLS, organized immune cell niches whose presence and maturation status have been associated with longer survival and improved response to ICI in various tumour types [22, 23, 43, 44]. To date, only two studies investigated the clinical relevance of TLS in PM: Mannarino et al. reported an increased presence of TLS in long-term survivors (>36 months) with epithelioid PM [45]. However, this study relied on conventional techniques such as immunohistochemistry and hematoxylin-eosin staining, which do not allow for the assessment of the contribution of different immune cells within the TLS and their maturation stages. Another study including 20 PM patients treated with neoadjuvant immunotherapy (anti-PD-L1 alone or combined with anti-CTLA-4) demonstrated that TLS presence in PM tumours significantly increased upon treatment and in patients with partial response [46]. Despite using advanced techniques like imaging mass cytometry, this study also lacked specific makers to differentiate between E-TLS, PFL-TLS and SFL-TLS.

In our study, we observed that patients in groups 1 and 2, who exhibited higher abundances of B cells and CD4^+^ T cells, showed a significant increase in TLS density compared to group 3. The functional and maturation state of TLS is a stronger predictor of favourable outcomes [23, 43]. In our study, further analysis of the different maturation stages revealed no significant differences in TLS densities at the different maturation stages between the three subgroups. However, a significant increase in absolute numbers of E-TLS was observed in groups 1 and 2 compared to group 3. Even though group 3 was represented by a small number of cases, our high-dimensional analysis allowed us to identify the immune cell subsets driving patients’ outcomes. While we present compelling results using a limited number of samples distributed among three different groups, it is necessary to validate these findings in a larger cohort of mesothelioma cases that can easily focus on major immune cell populations and TLS quantification and characterisation.

Recently, TLS induction has been actively explored as a strategy to enhance the efficacy of ICIs. Several studies have shown that conventional chemotherapies, particularly those inducing immunogenic cell death, along with immunotherapy, can promote TLS formation and improve patient outcomes [46–50]. Our results would support therefore the use of such therapies to induce TLS formation in patients with PM.

In conclusion, our study, using a limited number of patient samples, especially in group 3, highlights the importance of the characterisation of the TME of PM in guiding patients’ stratification and opening new perspectives for treatment development.

## Supplementary information

SupplementaryData
