# Supplementary material for: Multiplexed single-cell and spatial profiling reveal B cells and tertiary lymphoid structures as prognostic indicators in pleural mesothelioma
Source: Br J Cancer. 2026 Apr 27;135(2):214–22. doi: 10.1038/s41416-026-03421-1 (PMC13310852; doi:10.1038/s41416-026-03421-1)
Supplement: Supplementary file 1 — SupplementaryData [file 41416_2026_3421_MOESM1_ESM.pdf]

Supplementary data

Multiplexed Single-Cell and Spatial Profiling Reveal B Cells and Tertiary Lymphoid Structures as Prognostic Indicators in Pleural Mesothelioma

Rigutto A et al.

Supplementary Figures 1-4

Supplementary Tables 1-4

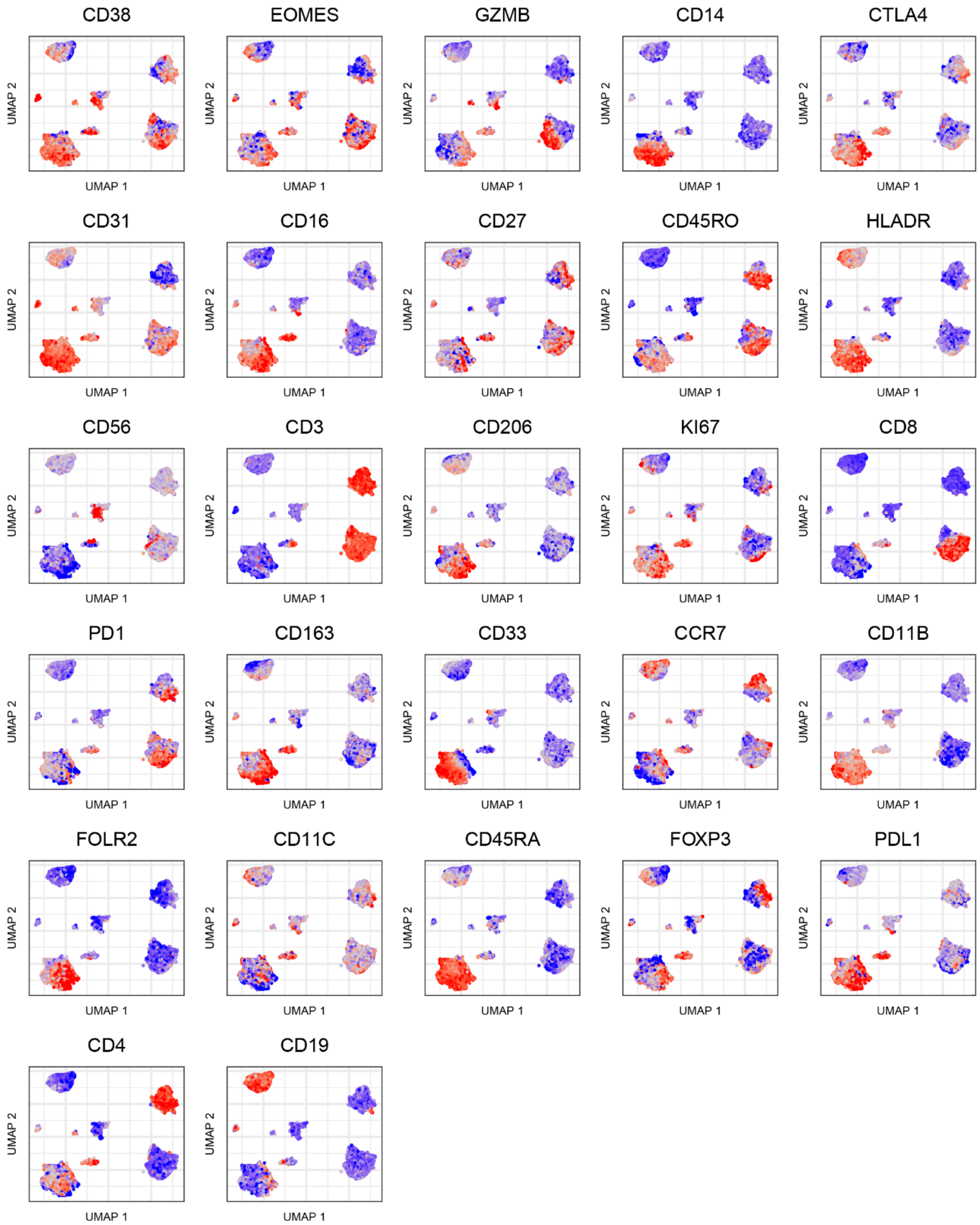

Supplementary Figure 1. UMAPs representing the marker intensities for each antibody used in the panel. These plots were used to manually annotate the immune cell populations.

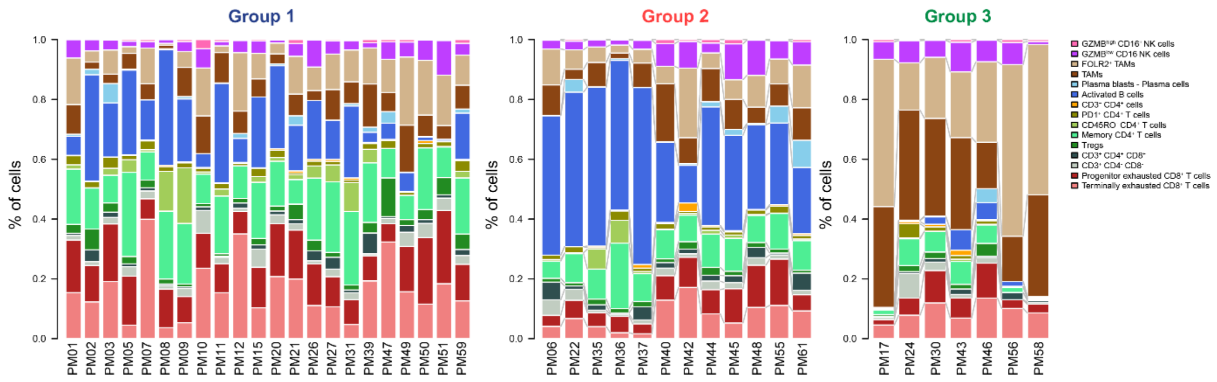

Supplementary Figure 2. Stacked bar plots representing the abundance of each immune cell subset in PM patients, divided by subgroups.

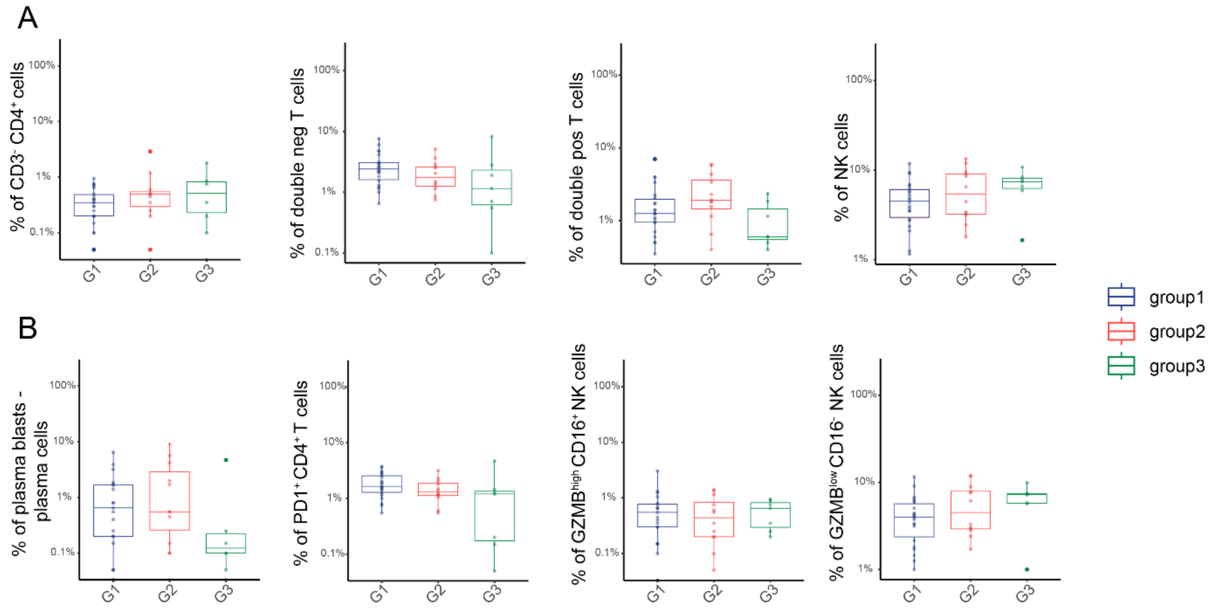

Supplementary Figure 3. Differential abundance analysis of major immune cell populations (A), and immune cell subsets (B) across the three subgroups (Group 1, n=22; Group 2, n= 12; Group 3, n=7). Non-significant results are reported in this figure. (Empirical Bayes quasi-likelihood F-test).

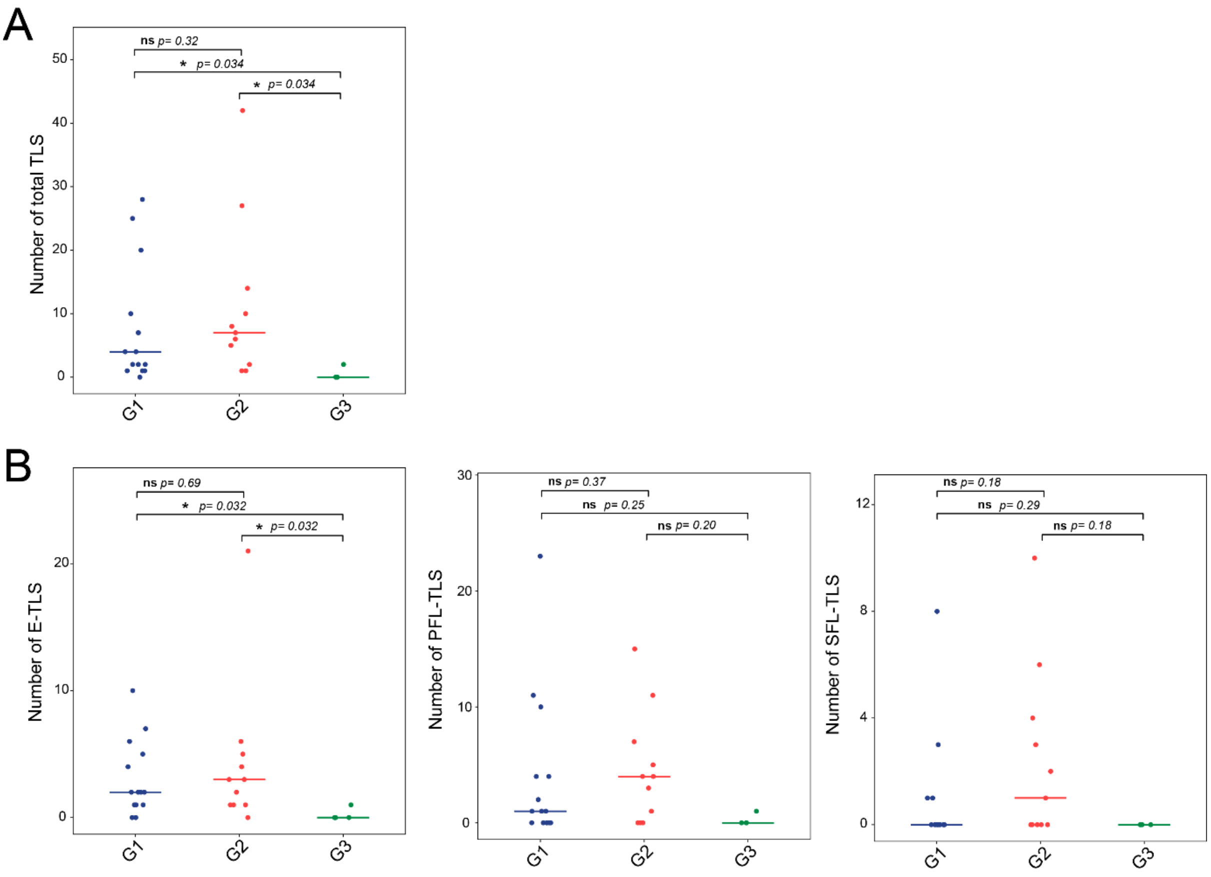

Supplementary Figure 4. Comparison of the absolute number of total TLS and of TLS at each maturation stage across subgroups (Group 1, n=15; Group 2, n= 11; Group 3, n=4. Wilcoxon-signed rank test). $p \leq 0.05$ (*), $p \leq 0.01$ (**), $p \leq 0.001$ (***), $p \leq 0.0001$ (****).

Supplementary Table 1. Antibodies panel.

HDCyto antibodies				
**Fluorophore**	**Antigen**	**Catalog Number**	**Company**	**Dilution**
PerCP	CD45	304026	BioLegend	1:100
BB770	CD14	745790	BD	1:100
BUV805	CD3	612895	BD	1:100
Spark Blue 550	CD4	344656	BioLegend	1:100
BV570	CD8	301038	BioLegend	1:100
BUV615	CD45RO	751167	BD	1:250
PE-Cy5.5	CD45RA	MHCD45RA18	ThermoFisher Scientific	1:200
BV785	CCR7	353230	BioLegend	1:100
BUV563	CD27	741366	BD	1:200
Super Bright 436	CD19	62-0199-42	ThermoFisher Scientific	1:75
BUV737	CD56	612766	BD	1:100
BUV496	CD16	612944	BD	1:150
BV421	CD206	321126	BioLegend	1:50
BUV395	CD31	565290	BD	1:100
Alexa Fluor 647	FAP	FAB3715R	R&D	1:200
BV650	CD163	563888	BD	1:50
PE-Cy5	CD11c	301609	BioLegend	1:150
APC-Fire 810	CD38	303550	BioLegend	1:100
BV711	CD33	303423	BioLegend	1:200
FITC	CD11b	301330	BioLegend	1:200
PE	FOLR2	391703	BioLegend	1:500
BUV661	HLADR	565073	BD	1:500
BV605	PD1	329924	BioLegend	1:100
PE/Dazzle™ 594	PDL1	563742	BD	1:500
BB630	MESOTHELIN	130-118-172	Miltenyi	1:100
Alexa Fluor 700	Granzyme B	560213	BD	1:250
BB790-P	CTLA-4	Custom	BD	1:50
APC-Cy7	Eomes	47-4877-42	BD	1:40
BV480	Ki67	566109	BD	1:100
PE-Cy7	FOXP3	25-4777-42	ThermoFisher Scientific	1:25
**High-plex imaging antibodies**				
Alexa Fluor 488	CD20	ab198941	Abcam	1:100
Alexa Fluor 647	Calretinin	ab21422	Abcam	1:500
Alexa Fluor 488	CD21	ab202692	Abcam	1:100
Alexa Fluor 647	CD4	ab196147	Abcam	1:100
Alexa Fluor 488	CD23	ab305270	Abcam	1:100

Supplementary Table 2. Survival analysis

**Group**	**adj. p.value**	**HR (95% CI)**
Overall survival (OS)		
G1	0.89	0.94 (0.44-2)
G2	0.22	0.59 (0.25-1.4)
G3	0.0014	13 (3.1-56)
Progression-free survival (PFS)		
G1	0.0645	0.44 (0.2-0.97)
G2	0.98	1 (0.42-2.4)
G3	0.00198	7.7 (2.8-22)

Univariate analysis

**Group**	**adj. p.value**	**HR (95% CI)**
Overall survival (OS)		
G1	0.1155	0.37 (0.15-0.93))
G2	0.41	1.2 (0.44-3.2)
G3	0.0054	21 (4.2-110))
Progression-free survival (PFS)		
G1	0.32	0.73 (0.32-1.7)
G2	0.32	0.68 (0.27-1.7)
G3	0.00237	42 (6.7-270)

Multivariate analysis

Supplementary Table 3. Differential abundance analysis of major immune cell populations and immune cell subsets across the three different subgroups.

MAJOR IMMUNE CELL POPULATIONS		
**Group 1 vs Group 2**		
Cell population	*Enriched group*	*Adj p-value*
$CD8^+$ T cells	Group 1	0.004
B cells	Group 2	0.005
$CD4^+$ T cells	Group 1	0.009
**Group 1 vs Group 3**		
Myeloid cells	Group 3	< 0.00001
$CD4^+$ T cells	Group 1	0.0007
B cells	Group 1	0.001
$CD8^+$ T cells	Group 1	0.004
**Group 2 vs Group 3**		
B cells	Group 2	< 0.00001
Myeloid cells	Group 3	0.00003
IMMUNE CELL SUBSETS		
**Group 1 vs Group 2**		
Cell population	*Enriched group*	*Adj p-value*
Terminally exhausted $CD8^+$ T cells	Group 1	0.017
Activated B cells	Group 2	0.017
Tregs	Group 1	0.017
Progenitor exhausted $CD8^+$ T cells	Group 1	0.017
Memory $CD4^+$ T cells	Group 1	0.020
**Group 1 vs Group 3**		
TAMs	Group 3	0.00001
$FOLR2^+$ TAMs	Group 3	0.0007
Memory $CD4^+$ T cells	Group 1	0.0008
Activated B cells	Group 1	0.002
$CD45RO^-$ $CD4^+$ T cells	Group 1	0.005
Progenitor exhausted $CD8^+$ T cells	Group 1	0.007
**Group 2 vs Group 3**		
Activated B cells	Group 2	< 0.00001
$FOLR2^+$ TAMs	Group 3	0.0001
TAMs	Group 3	0.0005
$CD45RO^-$ $CD4^+$ T cells	Group 2	0.02

Samples	age	gender	histology	Surgery	treatment_status	chemo_start	chemo_end	Cisplatin	Carboplatin	Pemetrexed	Bevacizumab	Response_to_treatment	OS	PFS
MPM01	68	male	Epithelioid	1	Post_chemo	06.09.2018	19.10.2018	1	0	1	1	SD	35.8	14
MPM02	65	male	Epithelioid	1	Post_chemo	18.09.2018	30.10.2018	0	1	1	0	SD	36.4	18
MPM03	68	male	Epithelioid	1	Post_chemo	26.09.2018	07.11.2018	1	0	1	1	PR	15.2	6
MPM05	71	male	Epithelioid	1	Post_chemo	05.03.2019	17.04.2019	0	1	1	1	SD	13.1	7
MPM06	79	male	Epithelioid	1	Post_chemo	21.03.2019	16.05.2019	0	1	1	1	SD	31.7	17
MPM07	67	male	Epithelioid	1	Post_chemo	03.04.2019	15.05.2019	1	0	1	0	PR	28	18
MPM08	67	male	Epithelioid	0	Pre_chemo	23.09.2019	02.12.2019	0	1	1	0	PR	24.6	19
MPM09	53	male	Epithelioid	1	Post_chemo	07.03.2019	27.06.2019	0	1	1	0	SD	16.3	1
MPM10	65	male	Epithelioid	1	Post_chemo	06.01.2019	01.5.2019	1	0	1	1	PR	31.9	9
MPM11	74	male	Epithelioid	0	Post_chemo	31.05.2019	11.7.2019	0	1	1	1	PR	23.7	NA
MPM12	64	male	Epithelioid	1	Post_chemo	29.05.2019	10.7.2019	1	0	1	1	PR	24	10
MPM13	74	male	Epithelioid	1	Post_chemo	31.05.2019	11.8.2019	0	1	1	1	PR	23.7	5
MPM14	70	male	Biphasic	0	NA	0	0	0	0	0	0	NA	1.3	
MPM15	66	male	Epithelioid	1	Post_chemo	22.07.2019	02.09.2019	1	1	1	0	PR	12.8	3
MPM16	65	male	Epithelioid	1	Post_chemo	18.08.2018	30.10.2018	0	1	1	0	SD	36.4	NA
MPM17	83	male	Epithelioid	0	Post_chemo	20.08.2019	01.10.2019	0	1	1	0	PD	6.3	2
MPM19	56	female	Epithelioid	0	Pre_chemo	06.1.2020	14.09.2020	0	1	1	1	SD	20.79	8
MPM20	68	male	Epithelioid	1	Post_chemo	01.9.2019	01.12.2019	1	0	1	1	PR	8.34	1
MPM21	61	male	Epithelioid	0	Pre_chemo	26.02.2020	08.4.2020	1	0	1	1	PR	20.99	9
MPM22	74	male	Epithelioid	0	Pre_chemo	02.3.2020	08.5.2020	0	1	1	1	PR	12.2	1
MPM24	76	male	Epithelioid	0	Pre_chemo	26.03.2020	08.06.2020	0	1	1	0	PR	14.6	2
MPM25	51	male	Biphasic	0	Post_chemo	11.5.2018	22.06.2018	0	1	1	1	PR	32.8	7
MPM26	58	male	Epithelioid	1	Post_chemo	28.10.2019	09.12.2019	0	1	1	0	SD	21.06	6
MPM27	60	male	Epithelioid	1	Post_chemo	13.01.2020	27.02.2020	1	0	1	1	NA	15.2	24
MPM28	74	male	Epithelioid	1	Post_chemo	02.03.2020	08.05.2020	0	1	1	1	PR	12.2	1
MPM29	61	male	Epithelioid	1	Post_chemo	26.02.2020	08.04.2020	1	0	1	1	PR	20.99	NA
MPM30	68	male	Epithelioid	0	Pre_chemo	0	0	0	0	0	0	NA	14.4	3
MPM31	80	male	Epithelioid	0	Pre_chemo	04.09.2020	0	0	1	1	1	PR	16.46	13
MPM33	68	male	Epithelioid	1	Pre_chemo	0	0	0	0	0	0	NA	14.4	3
MPM34	73	male	Epithelioid	0	Pre_chemo	27.08.2020	0	0	1	1	1	SD	5.72	6
MPM35	64	male	Epithelioid	1	Post_chemo	10.06.2020	22.08.2020	1	0	1	1	PR	15	7
MPM36	64	male	Epithelioid	1	Post_chemo	20.07.2020	04.09.2020	0	1	1	1	SD	7.8	7
MPM37	70	male	Epithelioid	1	Post_chemo	04.08.2020	15.09.2020	0	1	1	0	SD	14.39	NA
MPM39	77	male	Epithelioid	1	Post_chemo	08.07.2020	16.09.2020	0	1	1	0	PD	4.9	4
MPM40	73	male	Epithelioid	1	Post_chemo	12.11.2020	24.12.2020	0	1	1	1	SD	10.28	12
MPM42	66	male	Epithelioid	0	Pre_chemo	07.06.2021	20.07.2021	0	1	1	0	SD	6.97	4
MPM43	64	male	Biphasic	na	Post_chemo	29.01.2021	06.04.2021	1	0	1	0	PD	8.67	NA
MPM44	75	male	Epithelioid	0	Pre_chemo	0	0	0	0	0	0	PR	2.3	NA
MPM45	74	male	Epithelioid	1	Post_chemo	09.03.2021	24.04.2021	1	0	1	0	PR	11.43	5
MPM46	56	male	Epithelioid	0	Pre_chemo	25.06.2021	14.09.2021	1	0	1	1	PR	5.65	5
MPM47	73	male	Epithelioid	0	Pre_chemo	27.08.2020	0	0	1	1	1	NA	5.72	NA
MPM48	69	male	Epithelioid	1	Post_chemo	19.03.2021	28.04.2021	0	1	1	0	SD	5.45	10
MPM49	62	male	Epithelioid	1	Post_chemo	05.02.2021	28.05.2021	0	1	1	1	PR	7.65	11
MPM50	68	female	Epithelioid	1	Post_chemo	10.03.2021	27.05.2021	1	0	1	1	SD	10.02	6
MPM51	82	male	Biphasic	0	Pre_chemo	0	0	0	0	0	0	SD	3.84	NA
MPM52	82	male	Biphasic	1	Pre_chemo	0	0	0	0	0	0	SD	3.84	NA
MPM53	66	male	Epithelioid	1	Post_chemo	07.06.2021	20.07.2021	0	1	1	0	SD	6.97	4
MPM54	56	male	Epithelioid	0	Post_chemo	25.06.2021	14.09.2021	1	0	1	1	PR	5.65	5
MPM55	72	male	Epithelioid	1	Post_chemo	01.07.2021	03.09.2021	0	1	1	1	SD	8.41	NA
MPM56	64	female	Biphasic	0	Pre_chemo	01.11.2021	01.12.2021	0	1	1	0	PD	1.64	3
MPM57	70	male	Biphasic	0	Post_chemo	12.06.2020	27.07.2020	0	1	1	0	PR	15.18	4
MPM58	63	male	Epithelioid	0	Post_chemo	22.07.2021	24.09.2021	1	0	1	1	PD	1.12	3
MPM59	71	male	Epithelioid	1	Post_chemo	07.01.2020	28.02.2020	1	0	1	0	SD	23.85	9
MPM61	62	male	Epithelioid	0	Pre_chemo	30.11.2021	01.01.2022	0	1	1	0	SD	0.92	NA

Supplementary Table 4. Extended clinical data.
